# The Use of Premixed Calcium Silicate Bioceramic Sealer with Warm Carrier-Based Technique: A 2-Year Study for Patients Treated in a Master Program

**DOI:** 10.3390/jfb14030164

**Published:** 2023-03-18

**Authors:** Fausto Zamparini, Andrea Spinelli, Filippo Cardinali, Pietro Ausiello, Maria Giovanna Gandolfi, Carlo Prati

**Affiliations:** 1 Endodontic Clinical Section, Dental School, Department of Biomedical and Neuromotor Sciences, University of Bologna, 40125 Bologna, Italy; 2 Laboratory of Green Biomaterials and Oral Pathology, Dental School, Department of Biomedical and Neuromotor Sciences, University of Bologna, 40125 Bologna, Italy; 3 Private Practice, 60123 Ancona, Italy; 4 School of Dentistry, University of Naples Federico II, 80131 Naples, Italy; 5 Dean Master in Endodontics, Dental School, University of Bologna, Via San Vitale 59, 40125 Bologna, Italy

**Keywords:** carrier-based obturation, bioceramic sealers, calcium-silicate-based root canal sealer, calcium silicates, calcium silicate cements, bioceramics, periapical healing, apical extrusion, premixed CaSi, flowable CaSi, gutta-percha carrier endodontic

## Abstract

Background: Recently several calcium silicate flowable sealers have been introduced as endodontic materials for the root canal. This clinical study tested the use of a new premixed calcium silicate bioceramic sealer in association with the Thermafil warm carrier-based technique (TF). Epoxy-resin-based sealer with the warm carrier-based technique was the control group. Methodology: Healthy consecutive patients (n = 85) requiring 94 root canal treatments were enrolled in this study and assigned to one filling group (Ceraseal-TF n = 47, AH Plus-TF n = 47) in accordance with operator training and best clinical practice. Periapical X-rays were taken preoperatively, after root canal filling and after 6, 12 and 24 months. Two evaluators blindly assessed the periapical index (PAI) and sealer extrusion in the groups (k = 0.90). Healing rate and survival rate were also evaluated. Chi-square tests was used to analyze significant differences between the groups. Multilevel analysis was performed to evaluate the factors associated with healing status. Results: A total of 89 root canal treatments in 82 patients were analyzed at the end-line (24 months). The total drop-out was 3.6% (3 patients; 5 teeth). A total of 91.1% of healed teeth (PAI 1-2) was observed in Ceraseal-TF, with 88.6% in AH Plus-TF. No significant difference was observed on healing outcome and survival among the two filling groups (*p* > 0.05). Apical extrusion of the sealers occurred in 17 cases (19.0%). Of these, 6 occurred in Ceraseal-TF (13.3%) and 11 in AH Plus-TF (25.0%). Three Ceraseal extrusions were radiographically undetectable after 24 months. All the AH Plus extrusions did not change during the evaluation time. Conclusions: The combined use of the carrier-based technique and premixed CaSi-based bioceramic sealer showed clinical results comparable with carrier-based technique and epoxy-resin-based sealer. The radiographical disappearance of apically extruded Ceraseal is a possible event in the first 24 months.

## 1. Introduction

Calcium silicate cements (CaSi) were introduced in clinical use more than twenty years ago as root-end filling materials for their hydraulic properties [1,2].

Afterwards, thanks to the continuous modification of their chemical composition and mechanical properties, CaSi cements have been proposed as sealers for cold obturation techniques [3,4,5]. Recently, the formulation of these materials (now named “bioceramics”) changed from powder–liquid to premixed, flowable, “ready to use materials” [6,7,8,9,10]. Several CaSi-based (i.e., mainly containing CaSi particles) and CaSi-containing (i.e., containing minor amounts of CaSi) sealers are now available. 

Ceraseal is a premixed CaSi-based bioceramic sealer which includes tricalcium silicate (20–30%) and dicalcium silicate (1–10%) as bioactive components. Zirconium dioxide is the radiopacifier (45–50%) [11]. The chemical–physical properties have been investigated in only two studies [11,12]. These studies reported lower radiopacity, lower setting time and similar flowability when compared to AH Plus [11,12]. The ability to release calcium and to alkalize the environment was reported in one recent in vitro study [11]. These properties appear attractive for their clinical use in warm filling techniques, such as the warm carrier-based gutta-percha technique.

The clinical advantages of the warm carrier-based gutta-percha technique are well documented, including the easiness of the use, short learning curve, reliability in obtaining technical results [12,13,14,15] and adequate clinical results [15,16,17,18]. Traditionally, warm procedures such as carrier-based ones are associated with the use of epoxy-resin-based sealers [13]. Previous studies on carrier-based techniques reported success rates ranging from 81 to 96% after 3–5 years [15,16,18,19]. One of the drawbacks of the warm carrier-based technique is the higher risk of sealer extrusion in the periapical bone area [16,18]. In this context, the use of a CaSi-based bioceramic sealer may be clinically attractive in case of periapical extrusions. Clinical data on CaSi-based sealers used with cold filling techniques have been reported in recent short-term studies (12 months), reporting high percentages of success [20,21,22]. No clinical study reported the use of Ceraseal with warm carrier-based techniques. 

This clinical study analyzed the use of a new premixed CaSi-based bioceramic sealer in association with the warm carrier-based technique. Epoxy-resin-based sealer with the warm carrier-based technique was the control group. The null hypothesis was that both filling techniques and sealers provide a similar outcome after 24 months.

## 2. Materials and Methods

### 2.1. Study Design and Sample

This prospective cohort study was conducted between May 2019 and March 2022. The patients were treated in the Endodontic Clinical Section, Dental School, University of Bologna. Patients were treated by a pool of postgraduate master students in accordance with standardized protocols and under the strict supervision of the experienced tutors of the master degree. The study was approved by the local ethical committee as a prospective cohort study (OUTENDOPROSP; CE 20079).

All patients were treated according to the principles established by the Declaration of Helsinki as modified in 2013 [23]. Before enrolment, written and verbal information were given by the clinical staff and each patient gave their written consent according to the above-mentioned principles. An additional signed informed consent was obtained from all patients concerning the acceptance of the treatment plan and to follow the hygiene program. This study was designed according to the STROBE checklist [24] and to the guidelines published by Dodson in 2007 [25]. 

### 2.2. Sample Size 

The sample size was estimated before the recruitment of patients. The primary outcome was the survival status of teeth after root canal treatment with 2 different filling techniques. We assumed a 24-month survival rate of 0.80–0.90, according to previous studies [15,16]. Accepting a probability of a non-significant decrease in endodontic survival rate of 0.10 for patients whose teeth were filled with flowable premixed CaSi-based bioceramic sealer, we calculated that to test the null hypothesis of the equality of treatment at α = 0.05 with 80% power, a total of 40 teeth per group was considered sufficient. This number was increased to 47 per group (94 root canal treatments) to compensate for losses during follow-up.

Table 1 summarizes the criteria for the inclusion in the clinical protocol. Exclusion criteria are reported in Table 2.

**Figure 1:**
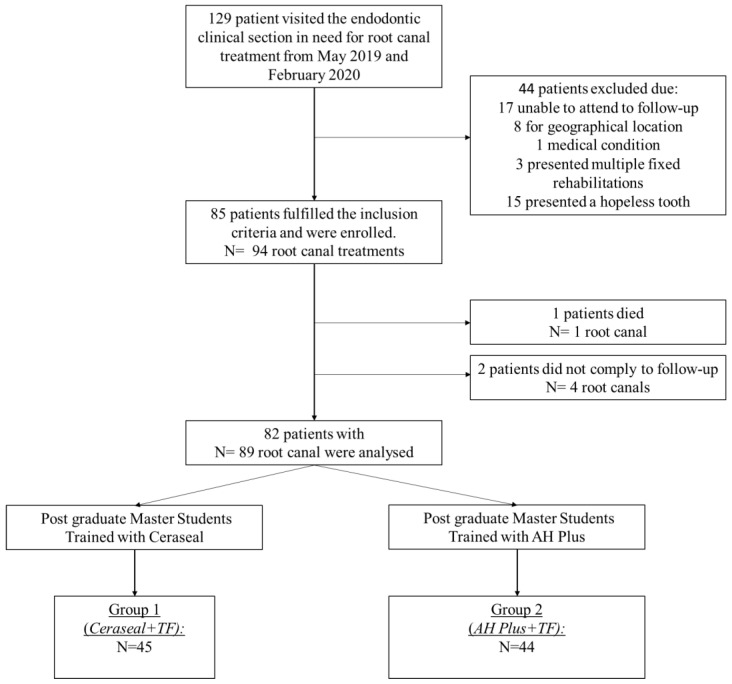
Flow chart of the study, patient enrolment and group constitution.

Patient enrolment started in May 2019 and ended in February 2020. During this period, 129 patients went in the endodontic clinical section in need of root canal treatment. Of these, 17 were excluded as unable to attend to regular follow-up examinations, 8 for geographical location (patients from other cities), 3 for presenting with multiple fixed rehabilitations, 1 for medical conditions, 15 for a tooth with insufficient structural integrity or for the tooth being considered hopeless (Figure 1).

### 2.3. Root Canal Treatment Procedures

Root canal treatments adhered to a standardized operative protocol. The procedures were made under local anesthesia (Carboplyne 30 mg/mL Dentsply, Germany) and rubber dam isolation.

For primary root canal treatment, a straight-line access was prepared using a diamond bur mounted on high-speed water-cooled hand-pieces (Cefla, Imola, Italy). Working length (WL) was determined at 0.5 mm from the radiographic apex with periapical radiographs and an electronic apex locator (Root ZX, Morita Europe, Dietzenbach, Germany). Root canals were first pre-flared with K-file instrumentation up to #15. Then, a series of NiTi instruments were used to shape the canals (Mtwo, VDW. Germany or Rotate, VDW, Munchen, Germany). During the instrumentation, 5 mL of 5% NaOCl (Niclor 5, OGNA, Muggiò, Italy) were used as root canal irrigant. In the presence of calcified root canals, 3.0 mL of 10% EDTA solutions was used as a calcium chelating agent. A final irrigation of 2.0 mL sterile water was made before root canal filling procedures [26,27,28]. No intracanal medication was placed in accordance with previous studies [26,29].

Secondary root canal treatments were performed using reciprocating NiTi instrumentation (Reciproc Blue, VDW, Munchen, Germany) and ultrasonic tips (StartX Dentsply Maillefer, Ballaigues, Switzerland). 

An initial pathway was created with ultrasonic tips to approximately 4 mm in depth in the gutta-percha. Reciproc Blue #25 was activated with a Silver Reciproc Endomotor (VDW, Germany) using the “Reciproc All” program and gently inserted into the pathway and pushed to remove the coronal part of gutta-percha [30]. The instrument was then removed, and the material entrapped among the instrument threads was removed using a sterile sponge. The working length was established at the apical constriction after the removal of filling debris using periapical radiographs and an electronic apex locator (Root ZX, Morita Europe, Dietzenbach, Germany).

Then, an apical enlargement was performed using Reciproc Blue #40 that was gently forced to the apex, avoiding excessive pressure on the root canal wall. 

During the instrumentation, 5 mL of 5% NaOCl (Niclor 5, OGNA, Muggiò, Italy) were used as the root canal irrigant. In the presence of calcified root canals, 3.0 mL of 10% EDTA solutions was used as a chelating agent. A final irrigation of 2.0 mL sterile water was made before the root canal filling procedures [26,27]. When necessary, a dental surgery microscope (Pico, Zeiss, Germany) was used to detect the access to root canal orifices and to identify the presence of residual filling materials.

### 2.4. Root Canal Filling Techniques and Filling Group Constitution

The premixed CaSi-based bioceramic sealer (Ceraseal, MetaBiomed, Cheongju, Republic of Korea) was used in association with the warm carrier-based technique (Thermafil, Dentsply, Konstanz, Germany). The sealer contained tricalcium silicate (20–30%), dicalcium silicate (1–10%), tricalcium aluminate (1–10%), zirconium dioxide (45–50%) and thickening agents [11]. Epoxy-resin-based sealer (AH Plus, Dentsply, Konstanz, Germany) in association with the warm carrier-based technique was used as a clinical control.

The root canal filling treatments were performed by 2 different operator groups previously trained for only one technique. Before the start of the study, (February–April 2019) post-graduate operators (n = 12) of the Master Program in Endodontics were randomly assigned to receive a full training course on the clinical use of the warm carrier-based technique with Ceraseal (Ceraseal-TF) or the use of the carrier-based technique with AH Plus (AH Plus-TF). 

In both filling groups, the carrier was pre-heated using a dedicated obturation oven (Thermaprep obturator oven, Dentsply, Konstanz, Germany)

*Ceraseal-TF* (*Ceraseal with Thermafil):* Ceraseal was applied with a K-file #20 inserted into the canal to reach the WL−3 mm and gently moved around the root canal walls. Pre-heated carrier was inserted in the canal at WL−0.5 mm. The carrier excess was removed with a round bur.

*AH Plus-TF (AH Plus with Thermafil)*: AH Plus was prepared, mixed and immediately inserted into the root canal using a K-file #20 to reach the WL−3 mm and gently moved around the root canal walls. Pre-heated carrier was inserted in the canal at WL−0.5 mm. The carrier excess was removed with a round bur.

Finally, each filled root canal was sealed by a small cotton pellet and a hygroscopic radiopaque temporary restoration (Coltosol, Coltene, Switzerland).

### 2.5. Tooth Restoration

The post-endodontic restoration protocol was similar in the 2 groups. Two weeks after root canal filling, the temporary restoration was removed using ultrasonic tips, and crown was restored under rubber dam isolation. Self-etching dentinal bonding agent primer and bonding (Clearfil SE BOND, Kuraray, Osaka, Japan) were applied, photo-cured (Elipar 3M ESPE, St. Paul, MN, USA) for 30 s and layered by flowable (G-Aenial Flow, GC Corporation, Tokyo, Japan) and composite (G-Aenial, GC Corporation) resins. The composite was incrementally applied with 1.0 mm layers and photo-cured for 30 s. All definitive restorations were occlusally loaded. No post was applied in any cases.

When indicated, provisional and definitive prosthetic rehabilitations were performed after approx. 6 months or later from root canal fillings. 

### 2.6. Radiological Evaluation 

Periapical radiographs were taken after the root canal filling using a parallel technique. The following parameters were used: the target-film distance was approx. 30 cm, with 0.41 s exposure at 70 kV and 8 mA. The radiographs were developed in a standard developer unit at 25 °C, (Euronda s.p.a., Vicenza, Italy), with 12 s developing time and 25 s fixing time, according to the manufacturer instructions. Patients were asked to undergo a new X-ray, when these characteristics were not fulfilled. 

The filling was considered “adequate” when the carrier was detected at 0–1.0 mm from the radiological apex. Overfilling and short filling were recorded. 

Each patient was monitored at 6 (T6), 12 (T12) and 24 (T24) months follow-up by trained operators during a routine hygiene appointment. X-rays were digitalized using a slide scanner with a mean resolution of 1000 dpi and a magnification factor of ×20.

The periapical index (PAI) [31] was used to score the preoperative diagnosis and the endpoint evaluations. PAI was evaluated in single blind by two additional operators (who did not participate in the root canal treatment) at baseline, at T6, T12 and T24 after root canal treatment. The presence of sealer extrusion was recorded and measured (in mm on the long axis diameter) on each periapical X-ray using open-source software (Image J, Bethesda, MD, USA). According to the long axis diameter, extrusions were categorized into 4 groups: 0 mm, 0.1–1.0 mm, 1.1–3.0 mm and >3 mm. 

PAI calibration was performed using well-defined instructions and periapical radiographs with different periapical lesion scores (weighted kappa value, k = 0.90 for intra-operator assessment and k = 0.90 for inter-operator assessment). 

### 2.7. Definition of Success and Survival Criteria

Teeth were defined as *healed* if they demonstrated no signs of periapical lesion (PAI 1-2) and no other clinical signs of symptoms (pain, mobility, fistula, swelling). These teeth defined the success rate [26].

The *endodontic lesion* group was defined when periapical radiolucency (PAI score ≥ 3) was detected during follow-up. A subdivision was made to discriminate between healing or stable lesions (improved or stable PAI during the follow-up) or worsened lesions (improved PAI during the follow-up)

The total of *healed* teeth and *endodontic lesion* teeth constituted the survival rate of the study [26]. The cause of extractions during the follow-up was recorded in a spreadsheet.

### 2.8. Statistical Analysis 

Analyses were performed using Stata 17 software (StataCorp LLC, College Station, TX, USA). Chi-squared and Fisher exact tests were used to evaluate differences in the parameter distribution between the 2 filling groups. Preoperative parameters were age (<30 vs. 30–55 vs. >55 years), gender (female vs. male), tooth location (anterior vs. premolar vs. molar) and pre-operative PAI (1-2 vs. 3-5). Intraoperative parameters were endodontic treatment (primary treatment vs. secondary treatment), obturation quality (underfilled vs. adequate filling vs. overfilled), sealer extrusion (yes/no) and diameter of extrusion (0 mm vs. 0.1–1.0 mm vs. 1.1–3.0 mm vs. >3.0 mm). The post-operative parameter was definitive restoration (composite vs. crown). Multilevel mixed-effect logistic regression was performed to determine the parameters related to healed status. This outcome measure was dichotomous as healed status included all teeth that did not present a periapical lesion after 24 months (PAI score 1-2) while the *endodontic lesion group* (non-healed) included all teeth that revealed a periapical lesion after 24 months (PAI score 3-5).

The analysis was carried out at patient and tooth level. Age and gender were set as person-specific fixed effects, while clinical parameters (filling technique, tooth location, diagnosis, initial PAI, endodontic treatment, obturation quality, sealer extrusion, diameter of extrusion and definitive restoration) were set as tooth-specific random effects. The clustering effect within patients was considered and estimates of coefficient standard errors were adjusted using a robust estimator. Statistical significance was previously set at 0.050. 

## 3. Results

### 3.1. Demographic Information

A total of 85 patients were initially enrolled with 94 root canal treatments. Total drop-out consisted of three patients with five root canal treatments. At the end-line, 82 patients with 89 root canal treatments were analyzed.

Information of preoperative, intraoperative and postoperative parameters between the two obturation groups is reported in Table 3. The two groups were homogeneous and did not significantly differ in almost all parameters (*p* > 0.05), with the only exception being age (*p* = 0.020, Ceraseal-TF included a higher percentage of filled teeth in patients younger than 30 years) and sealer extrusions (*p* = 0.040, Ceraseal-TF included a lower percentage of root canal teeth with sealer extrusions).

### 3.2. Outcome Measures

Cumulative 24-month survival rate was 97.8% as two teeth were lost (2.2%); one was filled with Ceraseal-TF and 1 with AH Plus-TF. The reasons for extraction were horizontal root fracture in both cases. No clinical manifestations, including pain, fistulae, or swelling were observed during the follow-up.

After 24 months, the percentage of healed teeth (teeth with no periapical lesion, PAI < 3) was 91.1% (Ceraseal-TF) and 88.6% (AH Plus-TF), respectively. No differences were observed between the groups (Pearson chi square = 0.9427, *p* = 0.624; Fisher exact test = 0.714) (Table 4).

Table 5 reports the comparison of healed status between the two filling groups according to the operative parameters. Some parameters showed differences in the percentages of healed teeth, such as tooth location, initial PAI, type of endodontic treatment, sealer extrusion, and diameter of extrusion (Table 5).

Multilevel-mixed logistic regression confirmed that only initial PAI and endodontic treatment parameters were statistically associated with a different healing outcome at 24 months (*p* < 0.05) (Table 6). A preoperative Initial PAI >3 and the presence of a previous endodontic treatment were predictors of a non-healing status at 24 months.

### 3.3. Sealer Extrusion and Sealer Resorption 

**Figure 2:**
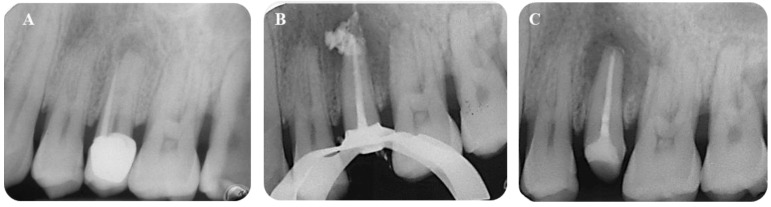
Ceraseal-TF group. (**A**) Tooth #25 presented a large periapical lesion (PAI 5) and a wide apical diameter (Ø 45). (**B**) Apical extrusion of the sealer occurred during the root canal filling procedures. (**C**) Apical extrusion was almost completely resorbed after 24 months.

**Figure 3:**
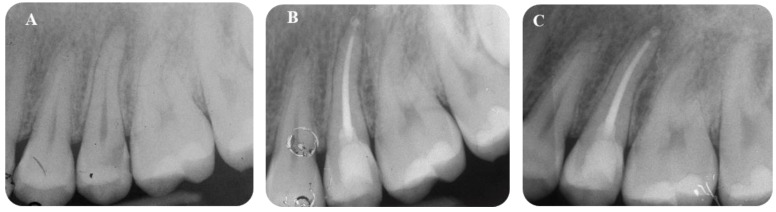
Ceraseal-TF group. (**A**) Tooth #25 was treated due to deep carious lesion and irreversible pulpitis. (**B**) Follow-up at 12 months from root canal filling (**C**) Note that apically extruded sealer showed a slight resorption after 24 months.

Table 7 reports the apically extruded sealer modifications in Ceraseal-TF and AH Plus-TF groups. Ceraseal-TF displayed 6 extrusions out of 44 root canal treatments (13.3%), while AH Plus-TF displayed 11 extrusions (25%). Three apical extrusions in the Ceraseal-TF group were resorbed after 24 months, and the other three were stable. Graphs reporting the sealer radiographical modification during follow-up are reported in Figure S1, Supplementary Materials. Representative periapical X-rays on apical extruded sealer modifications are reported in Figure 2 and Figure 3.

**Figure 4:**
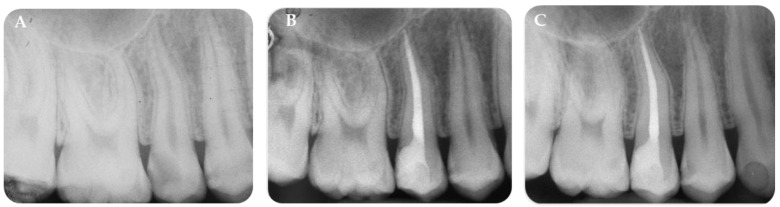
(**A**) Pre-operative periapical X-ray of a root canal treatment filled with AH Plus-TF group. Tooth #15 presented a deep caries lesion (PAI = 1). (**B**) No sealer extrusion was observed after root canal filling. (**C**) Follow-up at 24 months. Periapical area appears healthy with no signs of periapical exacerbation.

AH Plus-TF displayed 11 extrusions out of 44 root canal treatments (25%). The size and morphology of the resulting extrusion was stable and well-detectable after 24 months (Table 7 and Table S1, Supplementary Materials). One case of root canal treatment filled with AH Plus-TF is reported in Figure 4.

## 4. Discussion

This clinical study innovatively tested the use of a premixed CaSi-based bioceramic sealer with the warm carrier-based gutta-percha technique and demonstrated similar results when compared to epoxy-resin-based sealer used with the warm carrier-based techniques. Both filling procedures reported a high success rate (around 90%) after 24 months. 

In the present study, all the treatment procedures were conducted by post-graduate master operators after 3 months of specific training and under the strict supervision of the University Dental School tutors. The possibility of using warm carrier-based techniques in association with a premixed CaSi-based bioceramic sealer is useful and attractive for root canal obturation, as it combines the advantages of a carrier-based technique with the biological and bio-interactive properties of the sealer. 

CaSi materials showed innovative properties including the ability to set in a wet environment and to release biologically active ions [32,33,34], to expand [35,36,37] and to nucleate an apatite layer in phosphate-containing solutions [32,38,39,40,41]. Several studies reported a positive bio-interaction with periapical tissues and mineralizing cells [40,42,43,44] and osteoinductive properties with dynamic biomineralization processes [45].

These properties represent the rationale for the design of hydrophilic bio-interactive CaSi-based sealers and justify the expected good clinical outcome. 

A warm carrier-based technique was selected as it is widely considered the “gold standard” obturation technique [15,16,18,46]. Moreover, it is easier and more reproducible than other clinical techniques when proposed in a post-graduate master program, as reported in a large number of studies [15,16,18,46]. No previous studies clinically tested premixed CaSi-based bioceramic sealers with warm obturation techniques. The effect of heat application on CaSi-based sealers has been investigated in different in vitro studies [47,48,49]. Sealer dehydration and the degradation of organic components was observed on CaSi-based materials at high temperatures (100–225 °C degrees). Setting time, flowability [47,48,49] and film thickness [48] were critically affected. High temperatures may induce the thermal degradation of polyethyleneglycole (PEG), the water-soluble solvent included in the premixed sealer [49]. Actually, in vivo temperature is significantly lower than those reported in the previous studies. Donnermeier et al. demonstrated a maximum temperature rise to 58 °C when using warm-filling techniques [50]. Therefore, only limited modification of Ceraseal physical properties are expected when applied with a carrier-based technique. 

In the present study, the percentage of periapical healing increased from the initial preoperative status in both groups. The percentages of healed teeth (PAI 1-2), and the survival rate was similar in both groups. It is important to underline that no new periapical lesions or apical re-exacerbation was observed during the study.

Ceraseal-TF groups showed lower a percentage of periapical sealer extrusions (13.3%) when compared to the AH Plus-TF group (25%), which is likely attributable to the different chemical and physical properties of the two sealers. AH Plus used with warm techniques increase its fluidity and ability to flow out of the apex and penetrate deeper inside dentinal tubules [51]. Moreover, the radiopacity of AH Plus is markedly higher than Ceraseal (which is clinically evident from the periapical radiographs shown in Figure 3) and this may explain the high detectability and the greater diameter of the apically extruded AH Plus-TF.

It is known that the radiopacity of AH Plus is one of the highest among the current sealers, ranging from 10.00 mmAl to 11.8 mmAl [52,53]. Authors found Ceraseal radiopacity to be around 6.5 mmAl in a recent study [11]. The lower radiopacity of Ceraseal may require a greater volume of apical extrusion to be detected as a radiopaque mass in the periapical area.

In our study, three Ceraseal extrusions were resorbed and completely undetectable after 24 months. On the other hand, AH Plus extrusions proved stable results in all observational times. Our data on the stability of apically extruded AH Plus are in accordance with previous studies [54,55]. Limited information is reported regarding apical extrusion modification on CaSi-based sealers (and Ceraseal). Only two articles evaluated the frequency of apical over-extrusions of premixed CaSi-based bioceramic sealers [20,56]. Interestingly, both studies reported a higher apical over-extrusion of the premixed CaSi-based bioceramic sealers when compared to epoxy-resin-based sealer. Both studies considered cold filling techniques [20,56]. 

It should be underlined that the morphology and size of the extrusion is influenced by root diameter and most likely by the presence of a periapical lesion. Indeed, we found that the presence of periapical bone defects (observed in teeth with lesions PAI 3-5) were associated with sealer extrusions characterized by a circular morphology, while the (radiographic) integrity of apical bone that enveloped the apex was associated with a smaller extrusion (See Figure 2 and Figure 3). 

The effect of the periapical extrusion of the sealer on healing outcome has been debated in the literature. We would underline that the biological response to the extruded sealer strongly depends on the chemical composition of the material, on its release of biologically relevant ions or toxic components. A previous study reported that sealer extrusion lead to an unfavorable healing outcome as the sealer may act as a chronic source of inflammation in the periapical tissues [57]. More recent studies [54,55,58] found a not-significant effect of traditional extruded sealers on healing outcome.

We highlight that a different behavior in a biological environment must be supposed for Ceraseal and other premixed CaSi-based bioceramic sealers. The solubility of CaSi-based sealers was, in vitro, associated with high calcium release and was related with bioactivity (the ability to nucleate apatite), and with the consequent modification of the sealer structure in the apical region [32,39,43,59,60,61]. Animal models confirmed the bioactivity and the sealing ability of previous CaSi-based materials [62]. On the other hand, epoxy-resin-based sealers completely set and remained chemically and dimensional stable and inert in biological tissues, as demonstrated in a recent study [63]. 

The solubility of premixed CaSi-based sealers may be correlated with the (radiographic) disappearance of extruded sealers (Figure 2). The solubility of CaSi-based materials has been interlinked with their high calcium release [7,64,65]. The release of biologically active ions exerts a positive role in activating mineralizing stem cells [44,66]. CaSi components showed the ability to activate bone marrow cells [59], adipose-derived mesenchymal stem cells [67] and other cell types [68], in relationship with their biointeractive proprieties [32,43,44,45,59]. This could be clinically useful when the CaSi sealer is extruded into periapical bone defects or different bacteria-related bone resorptions.

Future studies are necessary to establish the long-term stability of the root canal seal, to verify if the sealer resorption also occurs along the root canal.

The use of NaOCl and EDTA as root canal irrigant solutions create a deep demineralized and deproteinized substrate [69] and increase dentin porosities and permeability, which are all conditions that may be responsible for root fracture. CaSi-based sealers can induce a new interfibrillar mineralization of demineralized dentine collagen, a mechanism described as dentin biomineralization [37,70,71,72]. Moreover, CaSi-based sealers showed, in vitro, their ability to penetrate into dentinal tubules of radicular dentine and to improve their sealing over time by filling potential interface voids [73] and by forming apatite [38] with associated dentine remineralization [38,70,71]. This is a further biological rationale in support of the use of CaSi-based sealers. 

In the present study, instrumentation techniques were similar in the two groups and adhered to a standardized operative protocol. Root canal instrumentation were performed with NiTi rotating systems—having a similar final diameter (0.25), taper (0.06) and cross section (S-shape)—in presence of primary root canal treatment, whereas a NiTi reciprocating system was used for secondary root canal treatment. 

A K-file #10 and 15 manual stainless system was used only for the initial preliminary scouting step and WL establishment. The technique was adopted to prevent any influence from the type of instrument. 

All post-graduate master students were skilled in the use of the rotary and reciprocating instruments. All efforts were made to follow the principles of best clinical practice. 

Preoperative tooth condition and a different initial diagnosis may influence root canal treatment healing (as reported in the multilevel analysis in Table 5). A long healing time increases the risks of apical reinfection in teeth affected by pulp necrosis or periapical abscesses [74,75,76], due to the presence of highly pathogenic Gram-negative anaerobic bacteria (as *Porphyromonas Gingivalis, Tannerella Forsithia, Prevotella Intermedia*) found in several clinical studies [76,77]. 

The study has some limitations including the short follow-up (24 months) and the prospective study design. A 24-month period may appear inadequate for the complete healing of teeth with a previous periapical lesion. ESE guidelines recommend a minimum period of 4 years to state the healing [78]. However, both groups showed a similar healing outcome and no periapical lesion re-exacerbation. 

We highlight that primary and secondary root canal treatments were not separately analyzed, leading to a potential bias in the presentation of the tooth survival results. Another limit could be considered the randomization of the operators. We decided to randomize the operators considering the importance in obtaining experience and confidence with the clinical use of premixed CaSi-based sealers, in particular with a warm technique. Therefore, we deemed it important to create a homogeneous group of trained operators. In this way, only the randomly selected operator was able enough for the treatment with a specific material and technique. Technical skills did not influence the clinical outcome, as no root canal perforations or iatrogenic complications occurred. Data on survival and success rate are high and in line with previously studies reporting an average success rate of 81–96% after 2–5 years [14,15,17,46]. For these reasons, the impact of the skills of post-graduate master students on the root canal treatment outcome was considered negligible. 

## 5. Conclusions

The study supports the clinical use of premixed CaSi-based sealer with the warm carrier-based technique. The two techniques showed a comparable clinical outcome. 

The study demonstrated that premixed CaSi-based sealer had less radiopacity, lower extrusion occurrence, but higher radiographic disappearance when apically extruded. This condition is likely in relationship with its solubility and calcium release. Interestingly, the sealer extrusion did not affect the healing outcome of root-canal-treated teeth.

Solid clinical scientific data may support the use of a flowable CaSi bioceramic sealer with a warm carrier-based gutta-percha technique and reduce some of the limits of epoxy-resin-based sealers. Further long-term studies may be important to validate these new filling materials.

## Figures and Tables

**Table 1:** Inclusion criteria.

✓ Aged 18–75 years
✓ Healthy status (ASA 1 or 2)
✓ No use of antiresorptive or antiangiogenic drug
✓ Needing one or more root canal treatments

**Table 2:** Exclusion criteria.

✓ ASA > 3
✓ Lack of occlusal contacts
✓ Heavy smoking (>15 cigarettes/day)
✓ Pregnancy or breast feeding
✓ Teeth with fewer than 2 walls of structural integrity
✓ Any pathology that could compromise bone healing or the immune response
✓ Malignant disease directly involving the jaws
✓ Exposure to radiation therapy focused on the head and neck region

**Table 3:** Demographic characteristics of root canal treatment followed-up in the study. Data expressed as number of root canal treatments (n) and their percentage (%) of the total sample. Bold indicates statistically significant differences (*p* < 0.05).

Parameters		Ceraseal-TF	AH Plus-TF	Chi Square Test
Age	<30 30–55 >55	15 (33.3) 12 (26.6) 18 (40.0)	7 (15.9) 19 (43.8) 18 (40.9)	Chi = 11.655 ***p* = 0.020**
Gender	Males Females	18 (40.0) 27 (60.0)	20 (45.4) 24 (54.6)	Chi = 0.810 *p* = 0.667
Tooth type	Incisors Canines Premolars Molars	10 (22.2) 2 (4.4) 11 (24.4) 22 (48.8)	4 (9.1) 2 (4.6) 10 (22.7) 28 (63.6)	Chi = 8.400 *p* = 0.078
Tooth location	Maxilla Mandible	27 (60.0) 18 (40.0)	29 (65.9) 14 (31.8)	Chi = 5.05 *p* = 0.080
Diagnosis	Prosthetic reasons Pulpitis Pulp Necrosis Re-exacerbated lesion	3 (6.6) 15 (33.3) 18 (40.0) 9 (20.0)	2 (4.6) 17 (38.6) 13 (29.5) 12 (27.2)	Chi = 15.672 *p* = 0.109
Initial PAI	PAI ≤2 PAI ≥3	19 (44.2) 26 (55.8)	21 (47.7) 23 (42.3)	Chi = 0.5332 *p* = 0.052
Endodontic treatment	Primary root treatment Secondary treatment	36 (80.0) 9 (20.0)	32 (72.2) 12 (27.8)	Chi = 1.05 *p* = 0.608
Obturation quality	Underfilled Adequate filling Overfilled	2 (4.4) 38 (84.4) 5 (11.1)	4 (9.1) 38 (86.3) 2 (4.6)	Chi = 3.287 *p* = 0.566
Sealer extrusion	No Yes	39 (86.6) 6 (13.4)	33 (75.0) 11 (25.0)	Chi = 6.451 ***p* = 0.040**
Long axis diameter of extrusion	0 0.1–1.0 mm 1.1–3.0 mm >3.0 mm	39 (84.4) 2 (4.4) 1 (2.2) 3 (6.6)	33 (75.0) 3 (6.8) 6 (13.6) 2 (4.5)	Chi = 5.53 *p* = 0.477
Definitive restoration	Composite Crown	36 (80.0) 9 (20.0)	37 (84.1) 7 (15.9)	Chi = 1.678 *p* = 0.416
Total		45	44	

**Table 4:** Percentage of healed, healing and extracted teeth after 24 months in the two groups. Data expressed as number of root canal treatments (n) and their percentage (%) of the total samples.

	n	Healed	Healing	Extracted
Ceraseal-TF	45	41 (91.1)	3 (6.8)	1 (2.3)
AH Plus-TF	44	39 (88.6)	4 (9.2)	1 (2.2)

**Table 5:** Comparison of healed status in the two filling groups in terms of pre-operative and intra-operative parameters. Data expressed as number of root canal treatments (n) and their percentage (%) of the total sample.

Parameters			Ceraseal-TF			AH Plus-TF	
		n	Healed	Healing	n	Healed	Healing
Age	<30	15	15 (100)	0 (0)	7	7 (100%)	0 (0)
	30–55	12	11 (91.6)	1 (8.4)	19	16 (84.2%)	3 (15.8)
	>55	17	15 (88.2)	2 (11.8)	17	16 (94.1%)	1 (5.9)
Gender	Males	17	16 (94.1)	1 (5.9)	19	18 (94.7%)	1 (5.3)
	Females	27	25 (92.5)	2 (7.5)	24	21 (87.5%)	3 (12.5)
Tooth type	Incisors	10	7 (70)	3 (30)	4	4 (100)	0 (0)
	Canine	2	2 (100)	0 (0)	2	2 (100)	0 (0)
	Premolars	10	10 (100)	0 (0)	10	9 (90)	1 (10)
	Molars	22	22 (100)	0 (0)	27	24 (88.8)	3 (11.2)
Tooth location	Maxilla	26	23 (88.4)	3 (11.6)	29	27 (93.1)	2 (6.9)
	Mandible	18	18 (100)	0 (0)	13	11 (84.6)	2 (15.4)
Initial PAI	PAI ≤2	18	18 (100)	0 (0)	20	20 (100)	0 (0)
	PAI ≥3	26	23 (88.4)	3 (11.6)	23	19 (82.6)	4 (17.4)
Diagnosis	Prosthetic reasons	3	3 (100)	0 (0)	2	2(100)	0 (0)
	Pulpitis	14	14 (100)	0 (0)	16	16 (100)	0 (0)
	Pulp necrosis	18	18 (100)	0 (0)	13	12 (92.3)	1 (7.7)
	Re-exacerbated lesion	9	6 (66.6)	3 (33.4)	12	9 (75)	3 (25)
Endodontic treatment	Primary root treatment	35	35 (100)	0 (0)	31	30 (96.7)	1 (3.3)
	Secondary treatment	9	6 (66.6)	3 (33.4)	12	9 (75)	3 (25)
Obturation quality	Underfilled	2	1 (50)	1 (50)	4	4 (100)	0 (0)
	Adequate	37	37 (100)	0 (0)	37	34 (91.8)	3 (8.2)
	Overfilled	5	5 (100)	0 (0)	2	1 (50)	1 (50)
Extrusion	No	38	37 (97.3)	1 (6.7)	32	29 (90.6)	3 (9.4)
	Yes	6	4 (66.6)	2 (33.4)	11	10 (90.9)	1 (9.1)
Long axis diameter of extrusion	0	38	37 (97.3)	1 (2.7)	32	29 (90.6)	3 (9.4)
	0.1–1.0 mm	2	2 (100)	0 (0)	3	3 (100)	0 (0)
	1.1–3.0 mm	1	1 (100)	0 (0)	6	5 (83.3)	1 (16.7)
	>3.0 mm	3	1 (33.3)	2 (66.7)	2	2 (100)	0 (0)
Definitive restoration	Composite	35	33 (94.2)	2 (5.8)	36	32 (88.8)	4 (11.2)
	Crown	9	8 (88.8)	1 (11.2)	7	7 (100)	0 (0)

**Table 6:** Multilevel-mixed logistic regression of parameters related to healed status after 24 months. Bold indicates significant values (*p* < 0.05).

Parameters		Robust		95% Confidence Interval	
	Coefficient	Standard Error	*p* Value	Lower Boundary	Upper Boundary
Intercept	1.488	0.201	0.000	1.0953	1.882
Age	−0.934	0.582	0.108	−2.075	0.206
Gender	−1.125	1.036	0.277	−3.156	0.905
Tooth location	0.643	0.542	0.235	−0.418	1.706
Diagnosis	0.044	0.715	0.951	−1.358	1.447
Initial PAI	−0.134	0.047	**0.005**	−0.228	−0.040
Endodontic treatment	−2.347	0.956	**0.014**	−4.216	−0.461
Filling technique	0.398	0.484	0.410	−0.551	1.348
Obturation quality	−1.60	0.917	0.081	−3.39	0.197
Sealer extrusion	0.410	0.995	0.680	−1.540	2.360
Diameter of extrusion	−0.099	0.064	0.124	−0.2271	0.027
Definitive restoration	−0.609	0.435	0.162	−1.463	0.244

**Table 7:** Modifications of apical extrusions at 24 months according to the two filling groups. Data expressed as number of root canal treatments (n) and their percentage (%) of the total sample.

	n	Stable	Resorbed	Total
Ceraseal-TF	45	3 (6.8)	3 (6.8)	6 (13.3)
AH Plus-TF	44	11 (25)	0 (0)	11 (25)

## Data Availability

The data presented in this study are available on request from the corresponding author. The data are not publicly available due to privacy and ethical reasons.

## Supplementary Materials

The following supporting information can be downloaded at: https://www.mdpi.com/article/10.3390/jfb14030164/s1, Figure S1: Graphs reporting the cases where sealer apical extrusion occurred. Periapical radiographs during the follow-up at 6, 12 and 24 months revealed the complete radiographical resorption of 3 extrusions in Ceraseal-TF group, one at 6 months and 2 at 12 months respectively. Differently, AH Plus-TF extrusions were stable along all the 24 months follow-up; Table S1: Quantification of radiographic apically extruded sealer in relation to PAI. Ceraseal +TF group displayed radiographic resorption of the sealer, while AH Plus+TF displayed no modification of extruded sealer.

## References

[1] Torabiinejad M., Chivian N. (1999). Clinical applications of mineral trioxide aggregate. J. Endod..

[2] Pitt-Ford T.R., Torabinejad M., McKendry D.J., Hong C.U., Kariyawasam S.P. (1995). Use of mineral trioxide aggregate for repair of furcal perforations. Oral Surg. Oral Med. Oral Pathol. Oral Radiol. Endod..

[3] Niu L.N., Jiao K., Wang T.D., Zhang W., Camilleri J., Bergeron B.E., Feng H.L., Mao J., Chen J.H., Pashley D.H. (2014). A review of the bioactivity of hydraulic calcium silicate cements. J. Dent. Res..

[4] Primus C.M., Tay F.R., Niu L.N. (2019). Bioactive tri/dicalcium silicate cements for treatment of pulpal and periapical tissues. Acta Biomater..

[5] Angerame D., De Biasi M., Pecci R., Bedini R. (2020). Filling ability of three variants of the single-cone technique with bioceramic sealer: A micro-computed tomography study. J. Mater. Sci. Mater. Med..

[6] Tanomaru-Filho M., Andrade A.S., Rodrigues E.M., Viola K.S., Faria G., Camilleri J., Guerreiro-Tanomaru J.M. (2017). Biocompatibility and mineralized nodule formation of Neo MTA Plus and an experimental tricalcium silicate cement containing tantalum oxide. Int. Endod. J..

[7] Zamparini F., Siboni F., Prati C., Taddei P., Gandolfi M.G. (2019). Properties of calcium silicate-monobasic calcium phosphate materials for endodontics containing tantalum pentoxide and zirconium oxide. Clin. Oral Investig..

[8] Giacomino C.M., Wealleans J.A., Kuhn N., Diogenes A. (2019). Comparative Biocompatibility and Osteogenic Potential of Two Bioceramic Sealers. J. Endod..

[9] López-García S., Myong-Hyun B., Lozano A., García-Bernal D., Forner L., Llena C., Guerrero-Gironés J., Murcia L., Rodríguez-Lozano F.J. (2020). Cytocompatibility, bioactivity potential, and ion release of three premixed calcium silicate-based sealers. Clin. Oral Investig..

[10] Hadis M., Camilleri J. (2020). Characterization of heat resistant hydraulic sealer for warm vertical obturation. Dent. Mater..

[11] Zamparini F., Prati C., Taddei P., Spinelli A., Di Foggia M., Gandolfi M.G. (2020). Chemical-Physical Properties and Bioactivity of New Premixed Calcium Silicate-Bioceramic Root Canal Sealers. Int. J. Mol. Sci..

[12] Kharouf N., Arntz Y., Eid A., Zghal J., Sauro S., Haikel Y., Mancino D. (2020). Physicochemical and Antibacterial Properties of Novel, Premixed Calcium Silicate-Based Sealer Compared to Powder-Liquid Bioceramic Sealer. J. Clin. Med..

[13] Goldberg F., Artaza L.P., De Silvio A. (2001). Effectiveness of different obturation techniques in the filling of simulated lateral canals. J. Endod..

[14] Mirfendereski M., Roth K., Bing F., Dubrowski A., Carnahan H., Azarpazhooh A., Basrani B., Torneck C.D., Friedman S. (2009). Technique acquisition in the use of two thermoplasticized root filling methods by inexperienced dental students: A micro-CT analysis. J. Endod..

[15] Pirani C., Zamparini F., Peters O.A., Iacono F., Gatto M.R., Generali L., Gandolfi M.G., Prati C. (2019). The fate of root canals obturated with Thermafil: 10-year data for patients treated in a master program. Clin. Oral Investig..

[16] Hale R., Gatti R., Glickman G.N., Opperman L.A. (2012). Comparative analysis of carrier-based obturation and lateral compaction: A retrospective clinical outcomes study. Int. J. Dent..

[17] Wong A.W.Y., Tsang C.S.C., Zhang S., Li S.K.Y., Zhang C., Chu C.H. (2015). Treatment outcomes of single-visit versus multiple-visit non-surgical endodontic therapy: A randomised clinical trial. BMC Oral Health.

[18] Demirci G.K., Caliskan M.K. (2016). A prospective randomized comparative study of cold lateral condensation versus Core/Gutta-percha in teeth with periapical lesions. J. Endod..

[19] Chu C.H., Lo E.C., Cheung G.S. (2005). Outcome of root canal treatment using Thermafil and cold lateral condensation filling techniques. Int. Endod. J..

[20] Chybowski E.A., Glickman G.N., Patel Y., Fleury A., Solomon E., He J. (2018). Clinical Outcome of Non-Surgical Root Canal Treatment Using a Single-cone Technique with Endosequence Bioceramic Sealer: A Retrospective Analysis. J. Endod..

[21] Zavattini A., Knight A., Foschi F., Mannocci F. (2020). Outcome of root canal treatments using a new calcium silicate root canal sealer: A non-randomized clinical trial. J. Clin. Med..

[22] Bardini G., Casula L., Ambu E., Musu D., Mercadè M., Cotti E. (2021). A 12-month follow-up of primary and secondary root canal treatment in teeth obturated with a hydraulic sealer. Clin. Oral Investig..

[23] World Medical Association (2013). World Medical Association Declaration of Helsinki: Ethical principles for medical research involving human subjects. J. Am. Med. Assoc..

[24] Vandenbroucke J.P., von Elm E., Altman D.G., Gøtzsche P.C., Mulrow C.D., Pocock S.J., Poole C., Schlesselman J.J., Egger M. (2007). STROBE Initiative Strengthening the Reporting of Observational Studies in Epidemiology (STROBE): Explanation and elaboration. Epidemiology.

[25] Dodson T.B. (2007). A guide for preparing a patient-oriented research manuscript. Oral Surg. Oral Med. Oral Pathol. Oral Radiol. Endod..

[26] Prati C., Pirani C., Zamparini F., Gatto M.R., Gandolfi M.G. (2018). A 20-year historical prospective cohort study of root canal treatments. A Multilevel analysis. Int. Endod. J..

[27] Zamparini F., Pelliccioni G.A., Spinelli A., Gissi D.B., Gandolfi M.G., Prati C. (2021). Root canal treatment of compromised teeth as alternative treatment for patients receiving bisphosphonates: 60-month results of a prospective clinical study. Int. Endod. J..

[28] Pirani C., Cirulli P.P., Chersoni S., Micele L., Ruggeri O., Prati C. (2011). Cyclic fatigue testing and metallographic analysis of nickel-titanium rotary instruments. J. Endod..

[29] Chong B.S., Ford T.R.P. (1992). The role of intracanal medication in root canal treatment. Int. Endod. J..

[30] Pirani C., Paolucci A., Ruggeri O., Bossù M., Polimeni A., Gatto M.R., Gandolfi M.G., Prati C. (2014). Wear and metallographic analysis of WaveOne and reciproc NiTi instruments before and after three uses in root canals. Scanning..

[31] Ørstavik D., Kerekes K., Eriksen H.M. (1986). The periapical index: A scoring system for radiographic assessment of apical periodontitis. Endod. Dent. Traumatol..

[32] Gandolfi M.G., Taddei P., Modena E., Siboni F., Prati C. (2013). Biointeractivity-related versus chemi/physisorption-related apatite precursor-forming ability of current root end filling materials. J. Biomed. Mater. Res. Part B Appl. Biomater..

[33] Rashid F., Shiba H., Mizuno N., Mouri Y., Fujita T., Shinohara H., Ogawa T., Kawaguchi H., Kurihara H. (2003). The effect of extracellular calcium ion on gene expression of bone-related proteins inhuman pulp cells. J. Endod..

[34] Sun J., Wei L., Liu X., Li J., Li B., Wang G., Meng F. (2009). Influences of ionic dissolution products of dicalcium silicate coating on osteoblastic proliferation, differentiation and gene expression. Acta Biomater..

[35] Gandolfi M.G., Iacono F., Agee K., Siboni F., Tay F., Pashley D.H., Prati C. (2009). Setting time and expansion in different soaking media of experimental accelerated calcium-silicate cements and ProRoot MTA. Oral Surg. Oral Med. Oral Pathol. Oral Radiol. Endod..

[36] Iacono F., Gandolfi M.G., Huffman B., Sword J., Agee K., Siboni F., Tay F., Prati C., Pashley D. (2010). Push-out strength of modified Portland cements and resins. Am. J. Dent..

[37] Gandolfi M.G., Taddei P., Siboni F., Modena E., Ginebra M.P., Prati C. (2011). Fluoride-containing nanoporous calcium-silicate MTA cements for endodontics and oral surgery: Early fluorapatite formation in a phosphate-containing solution. Int. Endod. J..

[38] Tay F.R., Pashley D.H., Rueggeberg F.A., Loushine R.J., Weller R.N. (2007). Calcium phosphate phase transformation produced by the interaction of the portland cement component of white mineral trioxide aggregate with a phosphate-containing fluid. J. Endod..

[39] Taddei P., Tinti A., Gandolfi M.G., Rossi P.L., Prati C. (2009). Vibrational study on the bioactivity of Portland cement-based materials for endodontic use. J. Mol. Struct..

[40] Gandolfi M.G., Taddei P., Tinti A., De Stefano Dorigo E., Prati C. (2011). Alpha-TCP improves the apatite-formation ability of calcium-silicate hydraulic cement soaked in phosphate solutions. Mater. Sci. Eng. C.

[41] Han L., Kodama S., Okiji T. (2015). Evaluation of calcium-releasing and apatite-forming abilities of fast-setting calcium silicate-based endodontic materials. Int. Endod. J..

[42] Rodríguez-Lozano F.J., López-García S., García-Bernal D., Tomás-Catalá C.J., Santos J.M., Llena C., Lozano A., Murcia L., Forner L. (2020). Chemical composition and bioactivity potential of the new Endosequence BC Sealer formulation HiFlow. Int. Endod. J..

[43] Hakki S.S., Bozkurt B.S., Ozcopur B., Gandolfi M.G., Prati C., Belli S. (2013). The response of cementoblasts to calcium phosphate resin-based and calcium silicate-based commercial sealers. Int. Endod. J..

[44] Gandolfi M.G., Shah S.N., Feng R., Prati C., Akintoye S.O. (2011). Biomimetic calcium-silicate cements support differentiation of human orofacial mesenchymal stem cells. J. Endod..

[45] Gandolfi M.G., Iezzi G., Piattelli A., Prati C., Scarano A. (2017). Osteoinductive potential and bone-bonding ability of ProRoot MTA, MTA Plus and Biodentine in rabbit intramedullary model: Microchemical characterization and histological analysis. Dent. Mater..

[46] Pirani C., Friedman S., Gatto M.R., Iacono F., Tinarelli V., Gandolfi M.G., Prati C. (2018). Survival and periapical health after root canal treatment with carrier-based root fillings: Five-year retrospective assessment. Int. Endod. J..

[47] Atmeh A.R., AlShwaimi E. (2017). The Effect of Heating Time and Temperature on Epoxy Resin and Calcium Silicate-based Endodontic Sealers. J. Endod..

[48] Yamauchi S., Watanabe S., Okiji T. (2020). Effects of heating on the physical properties of premixed calcium silicate-based root canal sealers. J. Oral Sci..

[49] Antunes T.B.M., Janini A.C.P., Pelepenko L.E., Abuna G.F., Paiva E.M., Sinhoreti M.A.C., Raimundo I.M., Gomes B.P.F.A., de-Jesus-Soares A., Marciano M.A. (2021). Heating stability, physical and chemical analysis of calcium silicate-based endodontic sealers. Int. Endod. J..

[50] Donnermeyer D., Schäfer E., Bürklein S. (2018). Real-time Intracanal Temperature Measurement during Different Obturation Techniques. J. Endod..

[51] Generali L., Cavani F., Serena V., Pettenati C., Righi E., Bertoldi C. (2017). Effect of Different Irrigation Systems on Sealer Penetration into Dentinal Tubules. J. Endod..

[52] Lee J.K., Kwak S.W., Ha J.H., Lee W., Kim H.C. (2017). Physicochemical Properties of Epoxy Resin-Based and Bioceramic-Based Root Canal Sealers. Bioinorg. Chem. Appl..

[53] Siboni F., Taddei P., Zamparini F., Prati C., Gandolfi M.G. (2017). Properties of BioRoot RCS, a tricalcium silicate endodontic sealer modified with povidone and polycarboxylate. Int. Endod. J..

[54] Ricucci D., Rôças I.N., Alves F.R., Loghin S., Siqueira J.F. (2016). Apically Extruded Sealers: Fate and Influence on Treatment Outcome. J. Endod..

[55] Goldberg F., Cantarini C., Alfie D., Macchi R.L., Arias A. (2020). Relationship between unintentional canal overfilling and the long-term outcome of primary root canal treatments and nonsurgical retreatments: A retrospective radiographic assessment. Int. Endod. J..

[56] Fonseca B., Coelho M.S., Bueno C.E.D.S., Fontana C.E., Martin A.S., Rocha D.G.P. (2019). Assessment of Extrusion and Postoperative Pain of a Bioceramic and Resin-Based Root Canal Sealer. Eur. J. Dent..

[57] Schaeffer M.A., White R.R., Walton R.E. (2005). Determining the optimal obturation length: A meta-analysis of literature. J. Endod..

[58] Aminoshariae A., Kulild J.C. (2020). The impact of sealer extrusion on endodontic outcome: A systematic review with meta-analysis. Aust. Endod. J..

[59] Gandolfi M.G., Ciapetti G., Perut F., Taddei P., Modena E., Rossi P.L., Prati C. (2009). Biomimetic calcium-silicate cements aged in simulated body solutions. Osteoblast response and analyses of apatite coating. J. Appl. Biomater. Biomech..

[60] Elsayed M.A., Hassanien E.E., Elgendy A.A.E. (2021). Ageing of TotalFill BC Sealer and MTA Fillapex in Simulated Body Fluid. Eur. Endod. J..

[61] Prati C., Gandolfi M.G. (2015). Calcium silicate bioactive cements: Biological perspectives and clinical applications. Dent. Mater..

[62] Reyes-Carmona J.F., Santos A.R., Figueiredo C.P., Felippe M.S., Felippe W.T., Cordeiro M.M. (2011). In vivo host interactions with mineral trioxide aggregate and calcium hydroxide: Inflammatory molecular signaling assessment. J. Endod..

[63] Jung S., Sielker S., Hanisch M.R., Libricht V., Schäfer E., Dammaschke T. (2018). Cytotoxic effects of four different root canal sealers on human osteoblasts. PLoS ONE.

[64] Siboni F., Taddei P., Prati C., Gandolfi M.G. (2017). Properties of NeoMTA Plus and MTA Plus cements for endodontics. Int. Endod. J..

[65] Razdan A., Benetti A.R., Bjørndal L. (2019). Do in vitro solubility studies on endodontic sealers demonstrate a high level of evidence? A systematic review. Acta Odontol. Scand..

[66] Matsumoto S., Hayashi M., Suzuki Y., Suzuki N., Maeno M., Ogiso B. (2013). Calcium ions released from mineral trioxide aggregate convert the differentiation pathway of C2C12 Cells into osteoblast lineage. J. Endod..

[67] Gandolfi M.G., Gardin C., Zamparini F., Ferroni L., Esposti M.D., Parchi G., Ercan B., Manzoli L., Fava F., Fabbri P. (2020). Mineral-Doped Poly(L-lactide) Acid Scaffolds Enriched with Exosomes Improve Osteogenic Commitment of Human Adipose-Derived Mesenchymal Stem Cells. Nanomaterials.

[68] Gandolfi M.G., Zamparini F., Esposti M.D., Chiellini F., Fava F., Fabbri P., Taddei P., Prati C. (2019). Highly porous polycaprolactone scaffolds doped with calcium silicate and dicalcium phosphate dihydrate designed for bone regeneration. Mater. Sci. Eng. C Mater. Biol. Appl..

[69] Gandolfi M.G., Taddei P., Pondrelli A., Zamparini F., Prati C., Spagnuolo G. (2019). Demineralization, Collagen Modification and Remineralization Degree of Human Dentine after EDTA and Citric Acid Treatments. Materials.

[70] Tay F.R., Pashley D.H. (2008). Guided tissue remineralisation of partially demineralised human dentine. Biomaterials.

[71] Tay F.R., Pashley D.H. (2009). Biomimetic remineralization of resin-bonded acid-etched dentin. J. Dent. Res..

[72] Gandolfi M.G., Taddei P., Siboni F., Modena E., De Stefano E.D., Prati C. (2011). Biomimetic remineralization of human dentin using promising innovative calcium-silicate hybrid “smart” materials. Dent. Mater..

[73] Gandolfi M.G., Parrilli A.P., Fini M., Prati C., Dummer P.M. (2013). 3D micro-CT analysis of the interface voids associated with Thermafil root fillings used with AH Plus or a flowable MTA sealer. Int. Endod. J..

[74] Love R.M., Jenkinson H.F. (2002). Invasion of dentinal tubules by oral bacteria. Crit. Rev. Oral Biol. Med..

[75] Ricucci D., Siqueira J.F. (2010). Biofilms and apical periodontitis: Study of prevalence and association with clinical and histopathologic findings. J. Endod..

[76] Foschi F., Izard J., Sasaki H., Sambri V., Prati C., Müller R., Stashenko P. (2006). Treponema denticola in disseminating endodontic infections. J. Dent. Res..

[77] Buonavoglia A., Zamparini F., Lanave G., Pellegrini F., Diakoudi G., Spinelli A., Lucente M.S., Camero M., Vasinioti V.I., Gandolfi M.G. (2023). Endodontic Microbial Communities in Apical Periodontitis. J. Endod..

[78] European Society of Endodontology (2006). Quality guidelines for endodontic treatment: Consensus report of the European Society of Endodontology. Int. Endod. J..

