# Supplementary material for: The Use of Premixed Calcium Silicate Bioceramic Sealer with Warm Carrier-Based Technique: A 2-Year Study for Patients Treated in a Master Program
Source: J Funct Biomater. 2023 Mar 18;14(3):164. doi: 10.3390/jfb14030164 (PMC10054578; doi:10.3390/jfb14030164)
Supplement: Supplementary file 1 [file jfb-14-00164-s001.zip › jfb-2141364-supplementary.pdf]

Supplementary Material

The Use of Premixed Calcium Silicate Bioceramic Sealer with Warm Carrier-Based Technique: A 2-Year Study for Patients Treated in A Master Program

Fausto Zamparini [1,2], Andrea Spinelli [1], Filippo Cardinali [3], Pietro Ausiello [4], Maria Giovanna Gandolfi [2] and Carlo Prati [1,5,*]

[1] Endodontic Clinical Section, Dental School, Department of Biomedical and Neuromotor Sciences, University of Bologna, Bologna, 40125, Italy
[2] Laboratory of Green Biomaterials and Oral Pathology, Dental School, Department of Biomedical and Neuromotor Sciences, University of Bologna, Bologna, 40125, Italy
[3] Private Practice, Ancona, 60123, Italy; filocardinali@gmail.com
[4] School of Dentistry, University of Naples Federico II, Naples, 80131, Italy
[5] Dean Master in Endodontics, Dental School, University of Bologna, Via San Vitale 59, 40125 Bologna, Italy
* Correspondence: carlo.prati@unibo.it

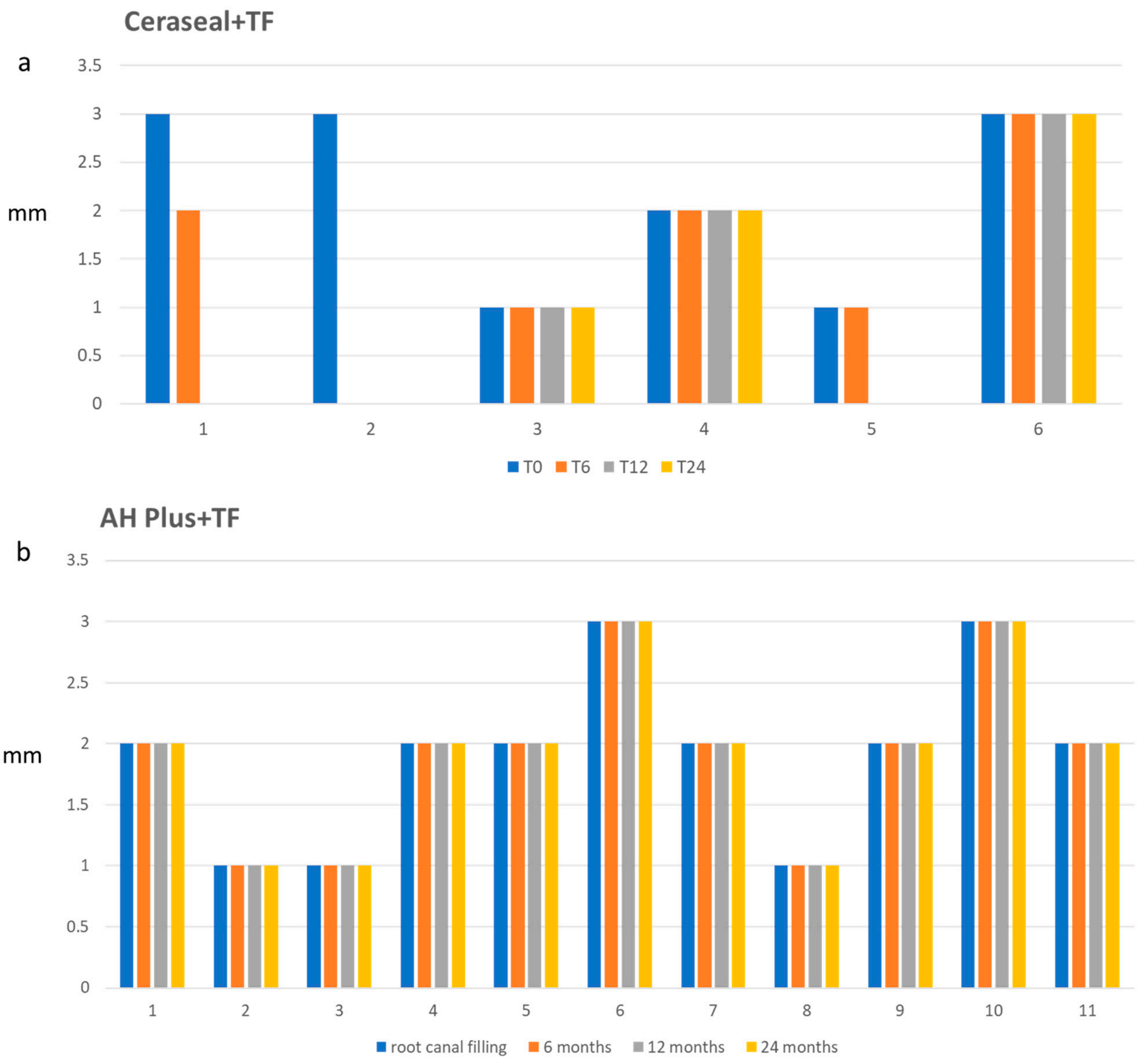

Figure S1. Graphs reporting the cases where sealer apical extrusion occurred. Periapical radiographs during the follow-up a t 6,12 and 24 months revealed the complete radiographical resorption of 3 extrusions in Ceraseal-TF group, one at 6 months and 2 at 12 months respectively. Differently, AH Plus-TF extrusions were stable along all the 24 months follow-up.

Table S1: Quantification of radiographic apically extruded sealer in relation to PAI. Ceraseal +TF group displayed radiographic resorption of the sealer, while AH Plus+TF displayed no modification of extruded sealer.

	**Ceraseal-TF**				**AH Plus-TF**			
	PAI 1-2		PAI 3-5		PAI 1-2		PAI 3-5	
	T0	T24	T0	T24	T0	T24	T0	T24
0mm	18	19	21	23	14	14	19	19
0.1-1mm	1	0	1	1	1	1	2	2
1.1-3mm	0	0	1	1	4	4	2	2
≥ 3mm	0	0	3	1	2	2	0	0
